# Supplementary material for: Population genomic analysis of an emerging pathogen Lonsdalea quercina affecting various species of oaks in western North America
Source: Sci Rep. 2023 Sep 8;13:14852. doi: 10.1038/s41598-023-41976-8 (PMC10491777; doi:10.1038/s41598-023-41976-8)
Supplement: Supplementary file 1 — Supplementary Information. [file 41598_2023_41976_MOESM1_ESM.pdf]

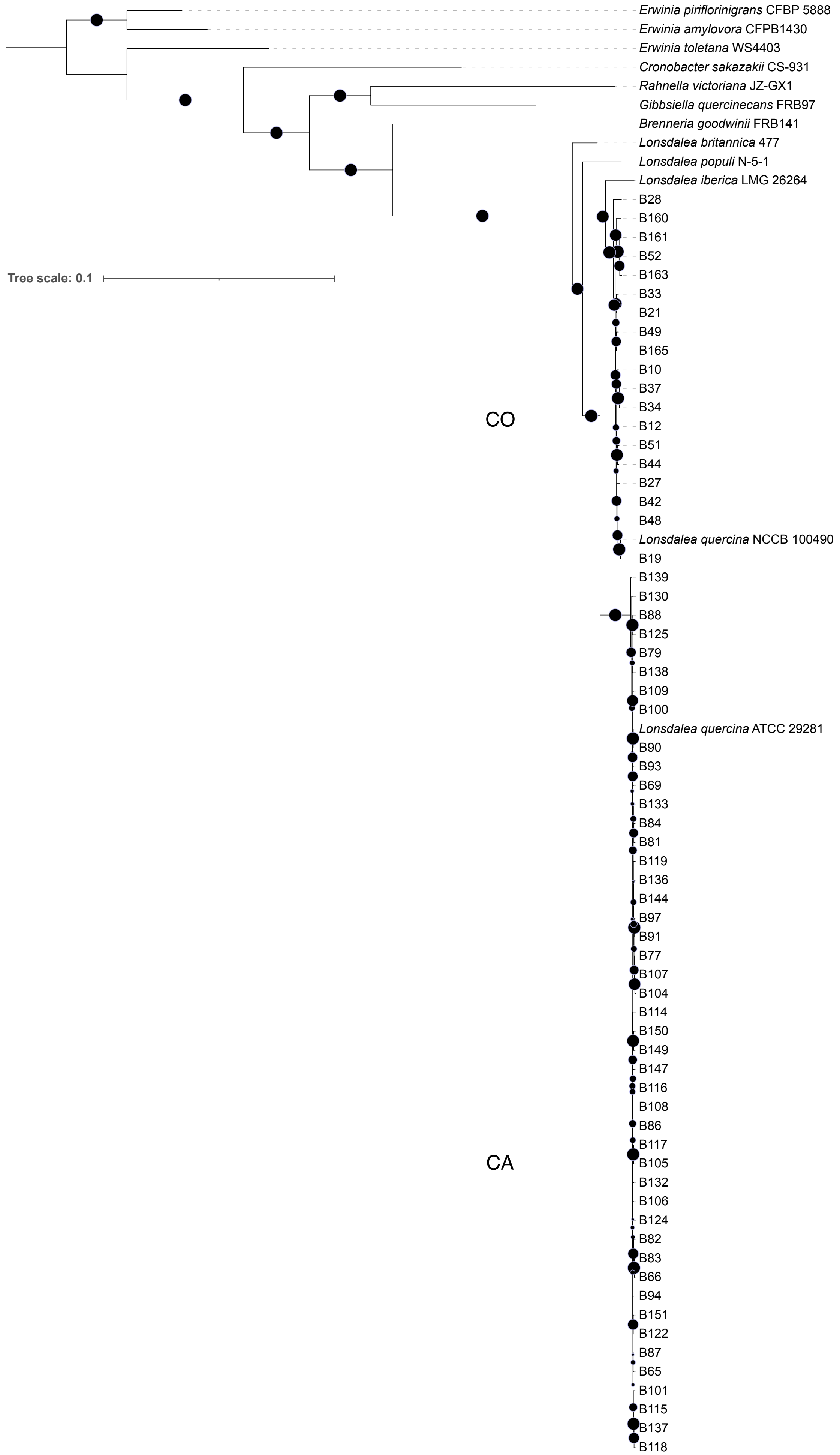

Figure S1. Maximum likelihood phylogenetic tree, based on 51 core genes shared among all individuals in the dataset, showing position of *Lonsdalea quercina* populations sampled in California (CA) and Colorado (CO), USA, within genus *Lonsdalea* and members from *Enterobacteriacea*, *Erwiniaceae*, *Pectobacteriaceae*, and *Yersiniaceae* families of the order *Enterobacterales* ord. nov. Branches with >=95% likelihood support are indicated with black circles. Branch support was calculated with 1000 ultrafast bootstrap replicates. *Erwinia piriflorinigrans* and *Erwinia amylovora* are used as a root.

a

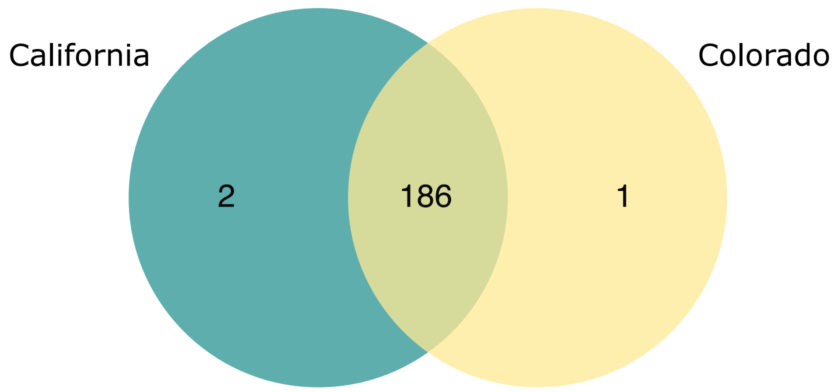

b

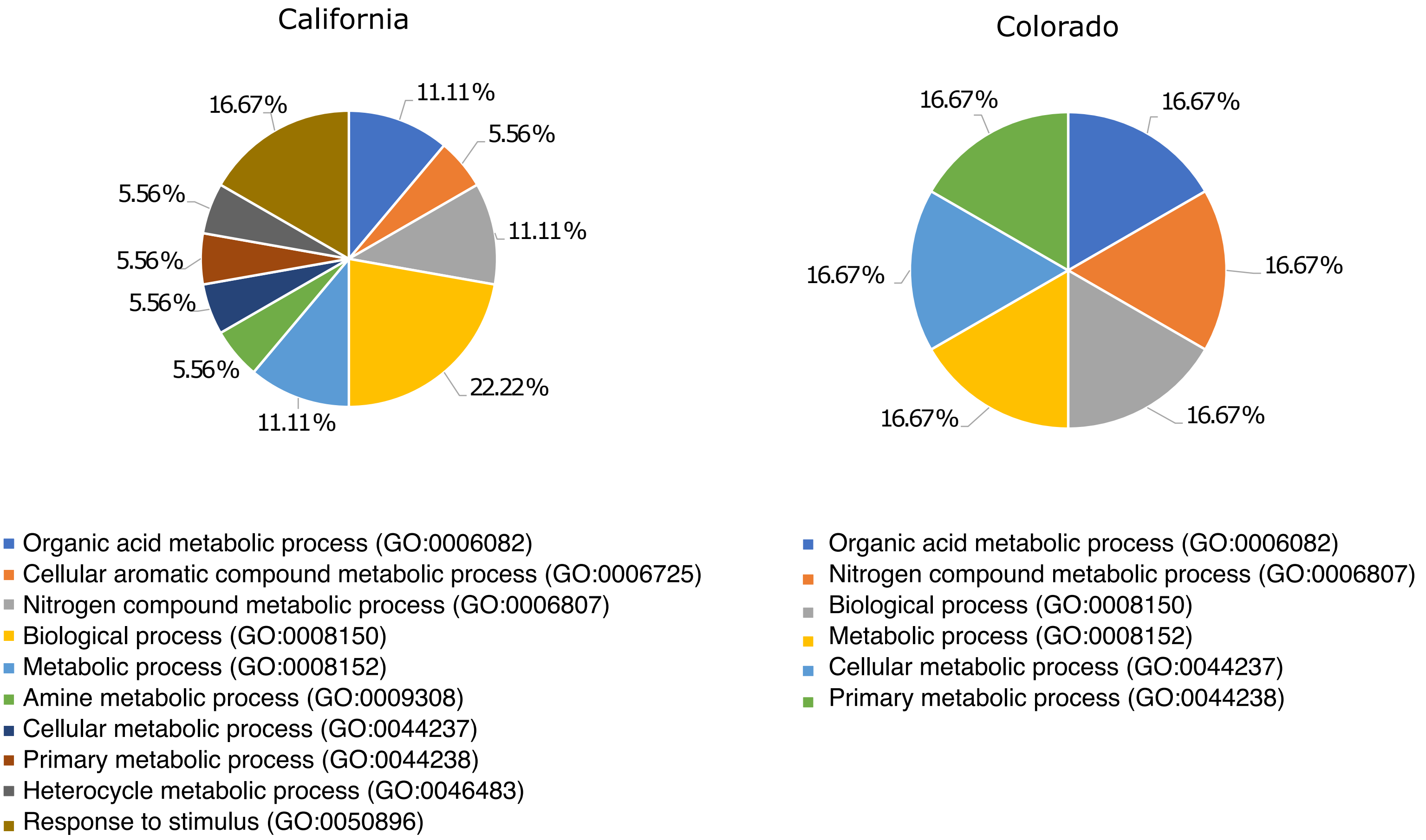

c

Enriched population specific GO annotations

Population	Cluster ID	GO ID	Name	# of proteins	P-value*
California	cluster 21	GO:0046690	response to tellurium ion	3	0.0002
Colorado	cluster 189	GO:0009086	methionine biosynthetic process	2	<0.0001

*P-value as reported by Orthevenn3, calculated via hypergeometric distribution.

d

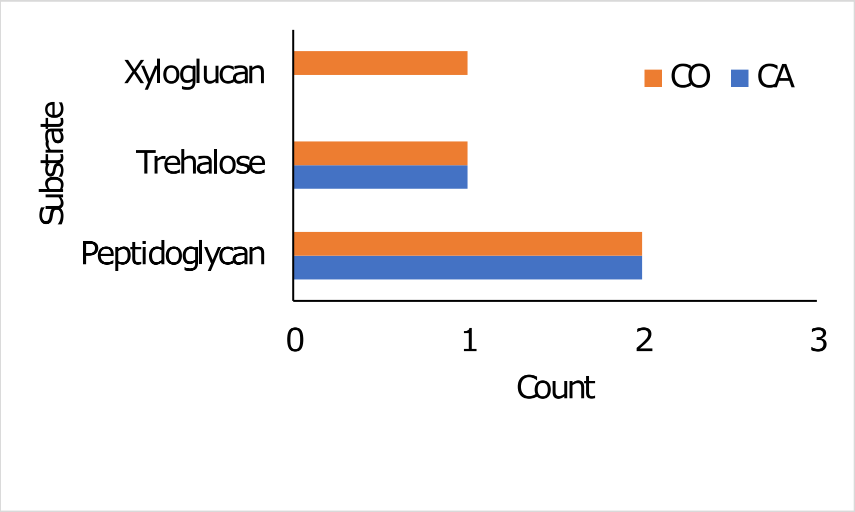

e

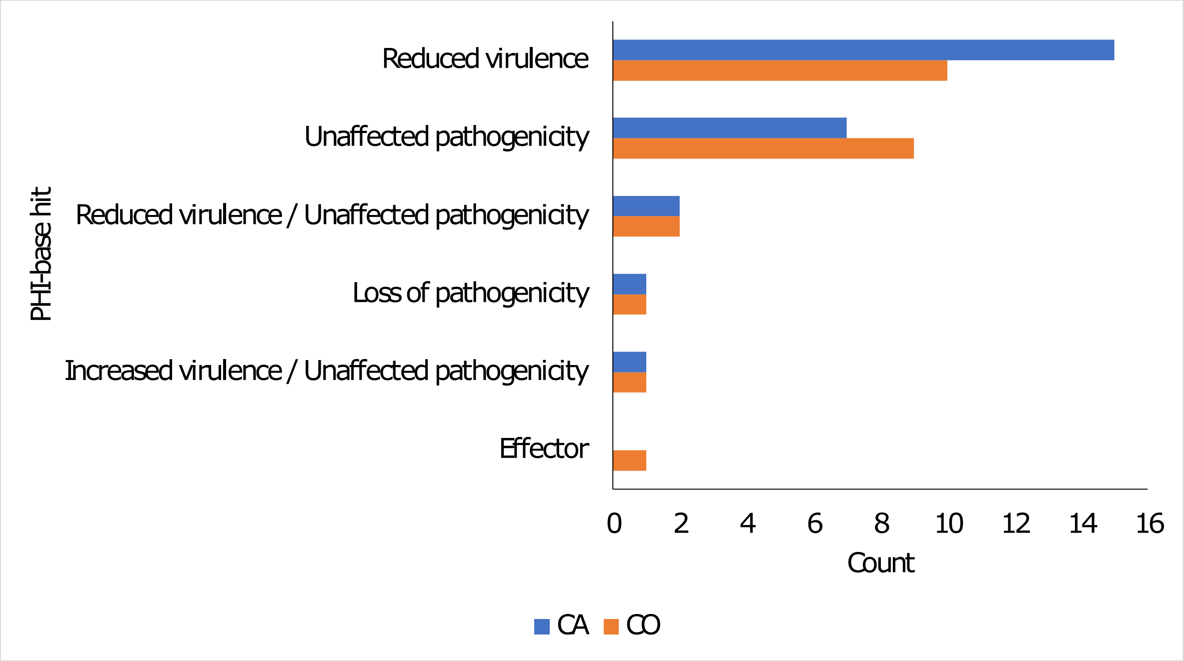

Figure S2. Results on protein annotations in California (CA) and Colorado (CO) populations of *Lonsdalea quercina*.
a. Venn diagram showing number of common and unique clusters identified in each population with Orthovenn3.
b. GO terms assigned to biological processes in each of the populations. **c.** Results of GO enrichment analyses for each of the populations. **(d)** Genes annotated as enzymes that degrade various substrates and **(e)** genes annotated as important for pathogenicity among 245 and 372 core genes unique to CA and CO *L. quercina* populations, respectively. Annotations were inferred from dbCAN2 and PHI-base, respectively. Detailed descriptions of annotations are given in Table S3 (dbCAN2) and Table S4 (PHI-base).

a

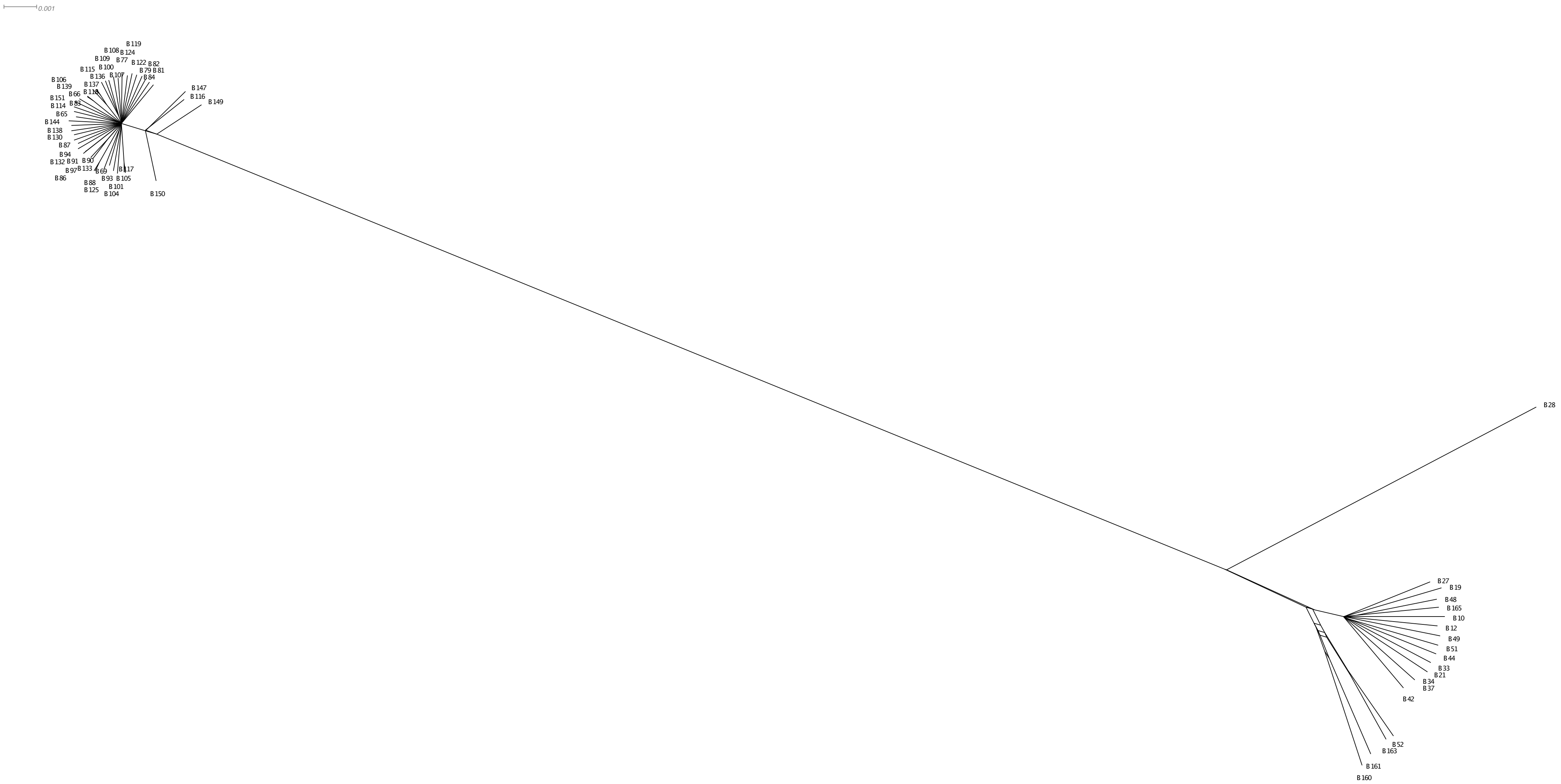
0.001
B 119
B 108
B 124
B 109
B 77
B 122
B 82
B 115
B 100
B 79
B 81
B 136
B 107
B 84
B 106
B 137
B 139
B 118
B 66
B 151
B 83
B 114
B 65
B 144
B 138
B 130
B 87
B 94
B 132
B 91
B 90
B 133
B 117
B 97
B 69
B 86
B 93
B 105
B 88
B 101
B 125
B 104
B 147
B 116
B 149
B 150
B 28
B 27
B 19
B 48
B 165
B 10
B 12
B 49
B 51
B 44
B 33
B 21
B 34
B 37
B 42
B 52
B 163
B 161
B 160

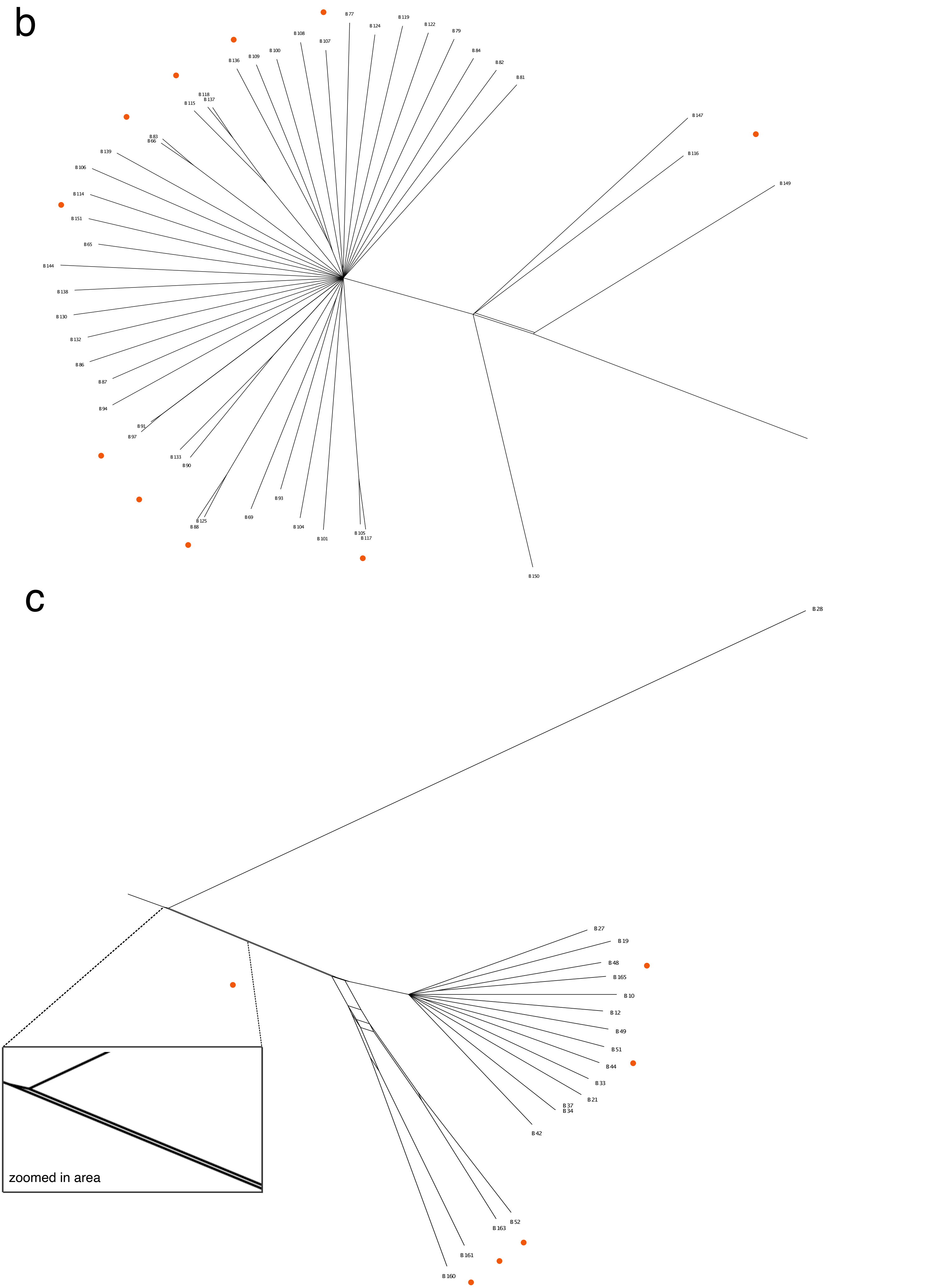

Figure S3. A SplitsTree analysis of recombination among and within CA and CO populations of *Lonsdalea quercina* (a). Enlarged clades of CA (b) and CO (c) populations from Fig.S3a. Red dots indicate recombination events detected by split decomposition analysis in SplitsTree.

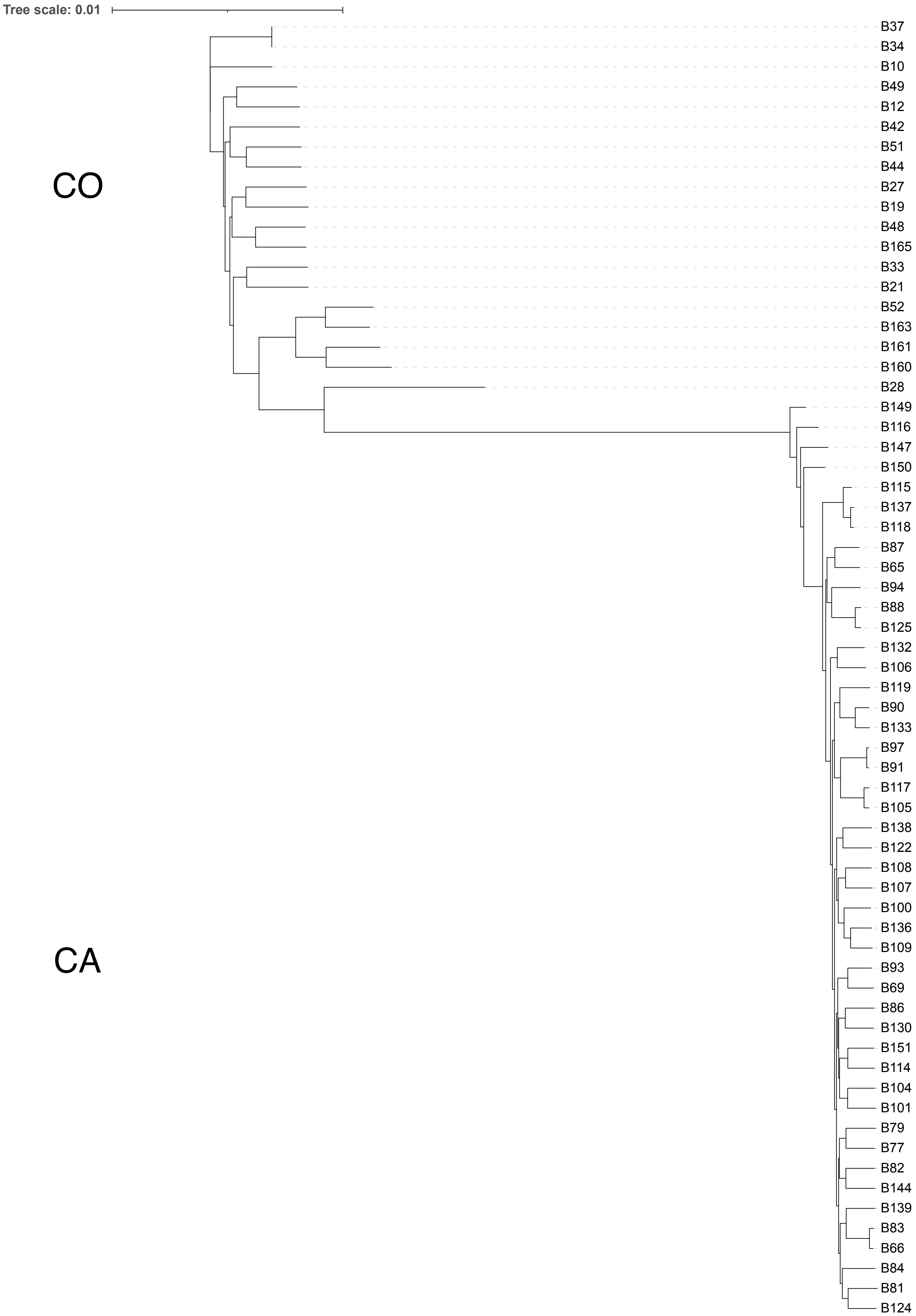

Figure S4. A phylogenetic tree of California (CA) and Colorado (CO) populations with branch lengths corrected toaccount for recombination produced by ClonalFrameML. A phylogenetic tree constructed with IQTREE v.2.0.3 (Fig.4a) was used as an input for this analysis.

Table S1. Isolates and genotypes list of *Lonsdalea quercina* collected in the study

Strain ID	State	Latitude	Longitude	Habitat*	Host	Genotype
B8	CO	40.01728	-105.261	Built	*Quercus rubra*	B12
B9	CO	40.01480	-105.251	Built	*Quercus rubra*	B19
B10	CO	40.01485	-105.256	Built	*Quercus rubra*	B10
B11	CO	40.01483	-105.258	Built	*Quercus rubra*	B10
B12	CO	40.01647	-105.261	Built	*Quercus rubra*	B12
B13	CO	40.01728	-105.261	Built	*Quercus rubra*	B12
B15	CO	40.01728	-105.261	Built	*Quercus rubra*	B10
B18	CO	40.01160	-105.276	Built	*Quercus rubra*	B10
B19	CO	40.00362	-105.285	Built	*Quercus rubra*	B19
B21	CO	40.00018	-105.284	Built	*Quercus rubra*	B21
B23	CO	40.00075	-105.272	Built	*Quercus rubra*	B19
B24	CO	40.00714	-105.261	Built	*Quercus rubra*	B19
B27	CO	39.72256	-105.171	Built	*Quercus rubra*	B27
B28	CO	39.99025	-105.091	Built	*Quercus rubra*	B28
B33	CO	39.71795	-104.894	Built	*Quercus rubra*	B33
B34	CO	39.71762	-104.895	Built	*Quercus rubra*	B34
B36	CO	39.72548	-104.902	Built	*Quercus rubra*	B34
B37	CO	39.72548	-104.902	Built	*Quercus rubra*	B37
B38	CO	39.72603	-104.886	Built	*Quercus rubra*	B33
B42	CO	39.72939	-104.962	Built	*Quercus rubra*	B42
B43	CO	39.72939	-104.962	Built	*Quercus rubra*	B42
B44	CO	39.72915	-104.984	Built	*Quercus rubra*	B44
B46	CO	39.73035	-104.992	Built	*Quercus rubra*	B33
B48	CO	39.75486	-105.006	Built	*Quercus rubra*	B48
B49	CO	39.73852	-104.981	Built	*Quercus rubra*	B49
B51	CO	39.73830	-104.949	Built	*Quercus rubra*	B51
B52	CO	39.73864	-104.910	Built	*Quercus palustris*	B52
B160	CO	39.34861	-104.764	Natural	*Quercus gambelii*	B160
B161	CO	39.34861	-104.764	Natural	*Quercus gambelii*	B161
B163	CO	39.29361	-104.922	Natural	*Quercus gambelii*	B163
B165	CO	38.85210	-104.786	Built	*Quercus rubra*	B165
B65	CA	38.26613	-122.178	Natural	*Quercus agrifolia*	B65
B66	CA	38.24700	-122.132	Built	*Qag*x*Qwis***	B66
B69	CA	38.49889	-122.296	Natural	*Qag*x*Qwis***	B69
B75	CA	38.29300	-122.472	Built	*Quercus agrifolia*	B125
B77	CA	38.37343	-122.517	Built	*Quercus agrifolia*	B77
B79	CA	38.46521	-122.657	Built	*Quercus agrifolia*	B79
B81	CA	38.39399	-122.894	Natural	*Quercus agrifolia*	B81
B82	CA	38.38899	-122.932	Natural	*Quercus agrifolia*	B82
B83	CA	38.46711	-123.011	Built	*Quercus agrifolia*	B83
B84	CA	38.07156	-122.540	Built	*Quercus agrifolia*	B84
B86	CA	38.06965	-122.539	Built	*Quercus agrifolia*	B86
B87	CA	38.02771	-122.568	Built	*Quercus agrifolia*	B87

B88	CA	37.94997	-122.522	Built	*Quercus agrifolia*	B88
B90	CA	37.59676	-122.419	Built	*Quercus agrifolia*	B90
B91	CA	37.59003	-122.412	Built	*Quercus agrifolia*	B91
B93	CA	37.32079	-122.275	Natural	*Quercus agrifolia*	B93
B94	CA	37.29810	-122.206	Natural	*Quercus parvula*	B94
B96	CA	37.25065	-121.968	Built	*Quercus agrifolia*	B107
B97	CA	37.21958	-121.977	Built	*Quercus agrifolia*	B97
B100	CA	36.38698	-121.550	Natural	*Quercus agrifolia*	B100
B101	CA	36.38522	-121.553	Natural	*Quercus agrifolia*	B101
B104	CA	36.37903	-121.567	Natural	*Quercus agrifolia*	B104
B105	CA	36.47806	-121.730	Built	*Quercus agrifolia*	B105
B106	CA	36.48193	-121.737	Built	*Quercus agrifolia*	B106
B107	CA	36.48753	-121.745	Built	*Quercus agrifolia*	B107
B108	CA	36.31391	-121.484	Built	*Quercus agrifolia*	B108
B109	CA	36.26679	-121.365	Natural	*Quercus agrifolia*	B109
B114	CA	35.29674	-120.661	Built	*Quercus agrifolia*	B114
B115	CA	35.91772	-120.684	Natural	*Quercus agrifolia*	B115
B116	CA	36.08174	-120.667	Natural	*Quercus agrifolia*	B116
B117	CA	36.52470	-121.144	Natural	*Quercus agrifolia*	B117
B118	CA	36.60729	-121.204	Natural	*Quercus agrifolia*	B118
B119	CA	38.07977	-122.254	Natural	*Quercus agrifolia*	B119
B120	CA	38.07775	-122.253	Natural	*Quercus agrifolia*	B100
B122	CA	38.07520	-122.257	Natural	*Quercus agrifolia*	B122
B124	CA	38.05130	-122.223	Built	*Quercus agrifolia*	B124
B125	CA	38.04881	-122.222	Built	*Quercus agrifolia*	B125
B127	CA	38.04826	-122.221	Natural	*Quercus agrifolia*	B122
B130	CA	38.04754	-122.221	Natural	*Quercus agrifolia*	B130
B132	CA	38.04708	-122.221	Natural	*Quercus agrifolia*	B132
B133	CA	38.03727	-122.210	Natural	*Quercus agrifolia*	B133
B136	CA	38.54288	-121.736	Built	*Quercus agrifolia*	B136
B137	CA	38.53661	-121.751	Built	*Quercus agrifolia*	B137
B138	CA	38.52952	-121.763	Built	*Quercus agrifolia*	B138
B139	CA	38.52959	-121.764	Built	*Quercus agrifolia*	B139
B140	CA	38.52934	-121.765	Built	*Quercus agrifolia*	B139
B144	CA	38.74962	-120.821	Natural	*Quercus wislizeni*	B144
B147	CA	39.22829	-121.141	Built	*Quercus wislizeni*	B147
B148	CA	39.20611	-121.272	Natural	*Quercus wislizeni*	B147
B149	CA	39.25365	-121.352	Natural	*Quercus wislizeni*	B149
B150	CA	39.34357	-121.336	Natural	*Quercus wislizeni*	B150
B151	CA	39.36149	-121.311	Natural	*Quercus wislizeni*	B151

*“Built” – an isolate from a tree located in a developed area (such as heavily developed city neighborhoods, parking lots etc.); “Natural” – an isolate from a tree located in undeveloped natural area (such as forest, nature preserve etc.)

***Qag*x*Qwis* – hybrid of *Q. agrifolia* and *Q. wislizeni*

Table S2. ANIb results for Colorado and California populations of *Lonsdalea quercina*

	B10	B12	B19	B21	B27	B28	B33	B34	B37	B42	B44	B48	B49	B51	B52	B160
**B10**	*	99.17	99.22	99.22	99.15	97.99	99.21	99.14	99.14	99.16	99.21	99.17	99.23	99.20	98.82	98.76
**B12**	99.28	*	99.27	99.26	99.28	98.05	99.25	99.27	99.28	99.28	99.27	99.27	99.29	99.26	98.91	98.83
**B19**	99.21	99.15	*	99.20	99.14	97.98	99.20	99.10	99.11	99.14	99.19	99.14	99.21	99.19	98.79	98.71
**B21**	99.26	99.24	99.24	*	99.23	98.02	99.27	99.22	99.22	99.22	99.23	99.23	99.27	99.24	98.89	98.80
**B27**	99.28	99.28	99.29	99.26	*	98.06	99.28	99.29	99.29	99.29	99.28	99.28	99.29	99.27	98.94	98.85
**B28**	98.05	98.03	98.04	98.03	98.03	*	98.03	98.03	98.03	98.04	98.04	98.03	98.05	98.03	97.90	97.91
**B33**	99.24	99.21	99.24	99.26	99.24	98.01	*	99.23	99.22	99.23	99.23	99.23	99.26	99.25	98.90	98.81
**B34**	99.28	99.29	99.27	99.27	99.29	98.06	99.28	*	99.91	99.29	99.28	99.29	99.29	99.27	98.93	98.85
**B37**	99.28	99.29	99.27	99.27	99.29	98.06	99.28	99.91	*	99.28	99.29	99.29	99.30	99.27	98.93	98.85
**B42**	99.28	99.28	99.27	99.26	99.28	98.05	99.27	99.27	99.28	*	99.28	99.29	99.30	99.26	98.92	98.83
**B44**	99.23	99.22	99.22	99.20	99.19	98.01	99.22	99.22	99.24	99.22	*	99.24	99.25	99.28	98.85	98.79
**B48**	99.27	99.27	99.27	99.26	99.27	98.05	99.28	99.26	99.28	99.28	99.29	*	99.28	99.27	98.91	98.83
**B49**	99.25	99.21	99.24	99.22	99.18	98.00	99.23	99.20	99.21	99.21	99.24	99.20	*	99.22	98.87	98.77
**B51**	99.24	99.22	99.23	99.22	99.20	98.01	99.24	99.21	99.23	99.22	99.30	99.22	99.26	*	98.88	98.79
**B52**	98.94	98.93	98.92	98.93	98.94	97.92	98.94	98.94	98.94	98.93	98.93	98.92	98.95	98.93	*	98.91
**B160**	98.83	98.80	98.81	98.81	98.84	97.91	98.83	98.83	98.83	98.82	98.83	98.81	98.84	98.81	98.89	*
**B163**	98.89	98.87	98.87	98.87	98.87	97.87	98.88	98.88	98.87	98.87	98.87	98.86	98.90	98.87	99.33	98.95
**B161**	98.82	98.81	98.81	98.81	98.84	97.89	98.84	98.82	98.82	98.83	98.82	98.82	98.83	98.82	98.99	99.16
**B165**	99.26	99.27	99.26	99.26	99.28	98.05	99.29	99.28	99.28	99.28	99.29	99.40	99.28	99.27	98.91	98.83
**B65**	95.16	95.13	95.16	95.16	95.14	94.84	95.17	95.12	95.11	95.14	95.16	95.12	95.17	95.16	95.08	95.06
**B66**	95.20	95.20	95.19	95.19	95.21	94.88	95.20	95.21	95.22	95.22	95.21	95.20	95.21	95.19	95.13	95.12
**B69**	95.19	95.18	95.18	95.18	95.17	94.87	95.19	95.19	95.20	95.19	95.19	95.18	95.19	95.17	95.11	95.10
**B77**	95.19	95.18	95.18	95.18	95.17	94.87	95.19	95.18	95.19	95.20	95.19	95.18	95.20	95.18	95.11	95.11
**B79**	95.21	95.21	95.20	95.20	95.22	94.90	95.21	95.22	95.22	95.23	95.22	95.21	95.22	95.20	95.14	95.13
**B81**	95.19	95.18	95.18	95.18	95.19	94.87	95.19	95.19	95.19	95.20	95.19	95.18	95.20	95.18	95.11	95.11
**B82**	95.20	95.20	95.20	95.19	95.21	94.89	95.20	95.22	95.22	95.22	95.20	95.20	95.21	95.19	95.13	95.12
**B83**	95.20	95.20	95.19	95.19	95.21	94.88	95.20	95.21	95.21	95.22	95.20	95.20	95.21	95.19	95.12	95.12
**B84**	95.20	95.19	95.19	95.19	95.18	94.87	95.20	95.19	95.20	95.21	95.20	95.19	95.20	95.18	95.12	95.11
**B86**	95.21	95.20	95.19	95.19	95.21	94.89	95.21	95.21	95.21	95.22	95.21	95.20	95.21	95.19	95.13	95.12
**B87**	95.16	95.13	95.15	95.15	95.13	94.83	95.16	95.12	95.12	95.14	95.16	95.13	95.16	95.13	95.06	95.06
**B88**	95.16	95.15	95.16	95.16	95.16	94.84	95.17	95.15	95.15	95.16	95.16	95.14	95.17	95.15	95.07	95.07
**B90**	95.20	95.19	95.19	95.19	95.20	94.88	95.20	95.20	95.21	95.21	95.20	95.19	95.20	95.19	95.13	95.12
**B91**	95.20	95.19	95.19	95.19	95.19	94.87	95.20	95.20	95.20	95.21	95.19	95.18	95.20	95.18	95.12	95.11
**B93**	95.21	95.20	95.20	95.20	95.22	94.88	95.21	95.22	95.22	95.22	95.21	95.20	95.21	95.19	95.13	95.13
**B101**	95.20	95.18	95.18	95.18	95.19	94.87	95.19	95.18	95.19	95.19	95.19	95.17	95.20	95.17	95.10	95.10
**B108**	95.19	95.19	95.19	95.18	95.18	94.88	95.20	95.20	95.20	95.21	95.19	95.18	95.20	95.18	95.12	95.11
**B147**	95.31	95.31	95.31	95.32	95.31	94.98	95.32	95.32	95.32	95.33	95.31	95.31	95.32	95.31	95.23	95.21
**B97**	95.18	95.15	95.17	95.16	95.16	94.85	95.17	95.17	95.17	95.18	95.17	95.15	95.18	95.16	95.09	95.09
**B104**	95.22	95.21	95.20	95.21	95.21	94.89	95.22	95.22	95.22	95.23	95.21	95.21	95.22	95.21	95.14	95.13
**B139**	95.20	95.19	95.19	95.18	95.20	94.87	95.20	95.19	95.20	95.20	95.19	95.19	95.20	95.18	95.12	95.11
**B144**	95.19	95.18	95.18	95.18	95.17	94.87	95.18	95.18	95.18	95.19	95.18	95.17	95.19	95.18	95.10	95.10
**B130**	95.19	95.18	95.18	95.18	95.18	94.87	95.19	95.18	95.19	95.20	95.19	95.18	95.20	95.18	95.10	95.10
**B137**	95.20	95.20	95.20	95.20	95.21	94.88	95.21	95.20	95.21	95.22	95.21	95.20	95.21	95.20	95.13	95.12
**B119**	95.18	95.16	95.18	95.16	95.17	94.85	95.17	95.15	95.16	95.17	95.17	95.15	95.18	95.16	95.10	95.09
**B107**	95.21	95.21	95.20	95.20	95.22	94.89	95.21	95.22	95.22	95.23	95.22	95.21	95.22	95.20	95.14	95.13
**B150**	95.28	95.28	95.27	95.29	95.28	94.95	95.29	95.29	95.30	95.30	95.28	95.27	95.29	95.27	95.20	95.18
**B94**	95.20	95.18	95.19	95.19	95.17	94.88	95.19	95.20	95.20	95.20	95.19	95.18	95.20	95.19	95.12	95.10
**B106**	95.20	95.18	95.19	95.19	95.18	94.87	95.20	95.18	95.19	95.20	95.20	95.18	95.20	95.19	95.11	95.11
**B115**	95.19	95.18	95.18	95.19	95.18	94.87	95.20	95.18	95.19	95.20	95.19	95.18	95.19	95.18	95.11	95.10
**B122**	95.19	95.18	95.19	95.18	95.20	94.87	95.19	95.18	95.17	95.20	95.19	95.17	95.20	95.18	95.11	95.10
**B132**	95.19	95.18	95.18	95.18	95.19	94.87	95.19	95.19	95.19	95.20	95.19	95.18	95.19	95.17	95.11	95.10
**B100**	95.19	95.18	95.18	95.18	95.20	94.87	95.19	95.18	95.17	95.19	95.19	95.17	95.19	95.18	95.11	95.10
**B138**	95.20	95.20	95.20	95.20	95.21	94.89	95.21	95.22	95.22	95.22	95.21	95.20	95.21	95.19	95.13	95.13
**B124**	95.20	95.20	95.19	95.19	95.19	94.88	95.20	95.20	95.20	95.20	95.20	95.19	95.21	95.19	95.13	95.11
**B109**	95.18	95.16	95.18	95.17	95.18	94.86	95.18	95.16	95.15	95.18	95.18	95.15	95.19	95.17	95.11	95.09
**B117**	95.20	95.20	95.19	95.19	95.20	94.89	95.20	95.21	95.21	95.21	95.20	95.19	95.21	95.19	95.12	95.12
**B116**	95.29	95.29	95.28	95.29	95.28	94.96	95.30	95.29	95.29	95.30	95.29	95.28	95.29	95.28	95.21	95.19
**B105**	95.20	95.20	95.19	95.19	95.20	94.89	95.20	95.21	95.21	95.21	95.20	95.20	95.21	95.19	95.12	95.12
**B133**	95.19	95.19	95.18	95.18	95.19	94.88	95.19	95.20	95.20	95.21	95.19	95.18	95.20	95.18	95.12	95.11
**B136**	95.19	95.17	95.18	95.17	95.19	94.86	95.19	95.17	95.17	95.18	95.18	95.16	95.19	95.17	95.11	95.09
**B118**	95.21	95.20	95.20	95.21	95.22	94.89	95.22	95.21	95.21	95.22	95.21	95.20	95.21	95.20	95.13	95.13
**B114**	95.20	95.18	95.19	95.18	95.19	94.87	95.20	95.18	95.18	95.19	95.20	95.17	95.20	95.19	95.10	95.10
**B125**	95.17	95.15	95.17	95.17	95.16	94.84	95.17	95.15	95.15	95.17	95.16	95.15	95.18	95.16	95.08	95.08
**B149**	95.35	95.34	95.34	95.35	95.34	95.00	95.36	95.35	95.35	95.36	95.35	95.34	95.35	95.34	95.26	95.25
**B151**	95.21	95.20	95.20	95.20	95.21	94.89	95.21	95.21	95.21	95.22	95.21	95.20	95.21	95.20	95.13	95.13

Table S2. Continued (page 2)

	B163	B161	B165	B65	B66	B69	B77	B79	B81	B82	B83	B84	B86	B87	B88	B90
**B10**	98.86	98.72	99.15	95.16	95.10	95.12	95.13	95.07	95.11	95.07	95.09	95.10	95.11	95.14	95.11	95.08
**B12**	98.91	98.81	99.27	95.20	95.19	95.19	95.20	95.18	95.19	95.17	95.19	95.19	95.20	95.19	95.18	95.18
**B19**	98.83	98.70	99.14	95.14	95.07	95.07	95.09	95.06	95.09	95.05	95.07	95.07	95.07	95.12	95.09	95.05
**B21**	98.88	98.79	99.23	95.20	95.17	95.18	95.20	95.15	95.18	95.14	95.17	95.19	95.19	95.19	95.18	95.16
**B27**	98.93	98.85	99.30	95.21	95.21	95.20	95.22	95.22	95.21	95.21	95.21	95.22	95.21	95.20	95.19	95.20
**B28**	97.89	97.89	98.02	94.87	94.86	94.87	94.89	94.85	94.86	94.85	94.86	94.86	94.87	94.87	94.86	94.86
**B33**	98.89	98.80	99.26	95.20	95.18	95.18	95.20	95.16	95.18	95.15	95.18	95.18	95.20	95.19	95.17	95.17
**B34**	98.93	98.84	99.29	95.21	95.21	95.21	95.22	95.22	95.21	95.21	95.21	95.22	95.21	95.21	95.20	95.20
**B37**	98.93	98.84	99.29	95.21	95.21	95.21	95.22	95.22	95.21	95.21	95.21	95.22	95.21	95.21	95.20	95.20
**B42**	98.91	98.84	99.28	95.21	95.21	95.21	95.22	95.20	95.20	95.20	95.21	95.21	95.22	95.21	95.19	95.21
**B44**	98.86	98.78	99.23	95.17	95.14	95.16	95.18	95.10	95.15	95.09	95.14	95.15	95.15	95.16	95.14	95.13
**B48**	98.91	98.83	99.40	95.20	95.19	95.20	95.21	95.18	95.19	95.17	95.19	95.20	95.20	95.19	95.18	95.19
**B49**	98.87	98.76	99.21	95.17	95.13	95.15	95.16	95.09	95.15	95.08	95.13	95.16	95.15	95.16	95.14	95.11
**B51**	98.87	98.78	99.23	95.17	95.15	95.15	95.17	95.12	95.16	95.11	95.14	95.15	95.15	95.15	95.14	95.13
**B52**	99.39	99.01	98.93	95.13	95.13	95.13	95.14	95.13	95.13	95.13	95.13	95.14	95.14	95.13	95.12	95.13
**B160**	98.97	99.15	98.81	95.11	95.11	95.11	95.12	95.09	95.11	95.08	95.11	95.11	95.12	95.11	95.09	95.10
**B163**	*	99.00	98.86	95.10	95.10	95.08	95.11	95.05	95.09	95.05	95.10	95.10	95.09	95.09	95.08	95.08
**B161**	99.04	*	98.82	95.13	95.12	95.13	95.14	95.11	95.12	95.10	95.12	95.14	95.14	95.13	95.12	95.12
**B165**	98.90	98.82	*	95.20	95.20	95.20	95.20	95.17	95.19	95.16	95.20	95.20	95.19	95.19	95.18	95.19
**B65**	95.08	95.06	95.13	*	99.48	99.50	99.49	99.41	99.48	99.44	99.48	99.49	99.48	99.56	99.51	99.49
**B66**	95.13	95.13	95.20	99.56	*	99.58	99.58	99.56	99.57	99.59	99.86	99.59	99.58	99.55	99.54	99.56
**B69**	95.11	95.11	95.18	99.54	99.55	*	99.54	99.52	99.54	99.54	99.54	99.55	99.55	99.53	99.52	99.55
**B77**	95.11	95.11	95.17	99.53	99.53	99.53	*	99.50	99.53	99.50	99.53	99.56	99.51	99.52	99.50	99.52
**B79**	95.14	95.14	95.21	99.52	99.57	99.56	99.56	*	99.54	99.55	99.58	99.56	99.56	99.52	99.52	99.54
**B81**	95.11	95.11	95.18	99.54	99.53	99.54	99.54	99.49	*	99.52	99.53	99.55	99.53	99.53	99.52	99.54
**B82**	95.13	95.13	95.20	99.55	99.59	99.57	99.55	99.55	99.57	*	99.59	99.58	99.58	99.54	99.53	99.56
**B83**	95.12	95.13	95.19	99.56	99.86	99.57	99.57	99.57	99.56	99.58	*	99.59	99.58	99.55	99.54	99.56
**B84**	95.12	95.12	95.19	99.56	99.55	99.55	99.56	99.51	99.55	99.53	99.55	*	99.53	99.54	99.52	99.53
**B86**	95.13	95.13	95.19	99.55	99.58	99.58	99.56	99.55	99.57	99.57	99.58	99.57	*	99.54	99.53	99.56
**B87**	95.07	95.06	95.13	99.56	99.47	99.49	99.48	99.40	99.47	99.42	99.47	99.47	99.47	*	99.51	99.47
**B88**	95.09	95.08	95.14	99.53	99.49	99.49	99.49	99.42	99.49	99.44	99.49	99.49	99.47	99.53	*	99.50
**B90**	95.12	95.13	95.19	99.57	99.56	99.57	99.55	99.53	99.56	99.55	99.56	99.56	99.56	99.55	99.54	*
**B91**	95.12	95.12	95.18	99.54	99.55	99.54	99.53	99.54	99.55	99.56	99.54	99.54	99.54	99.53	99.52	99.55
**B93**	95.13	95.14	95.20	99.55	99.60	99.62	99.57	99.55	99.56	99.59	99.59	99.59	99.59	99.55	99.54	99.57
**B101**	95.12	95.11	95.17	99.52	99.53	99.54	99.55	99.48	99.53	99.50	99.53	99.55	99.54	99.52	99.49	99.52
**B108**	95.12	95.12	95.18	99.54	99.55	99.56	99.54	99.53	99.55	99.54	99.55	99.55	99.54	99.55	99.53	99.55
**B147**	95.23	95.22	95.30	99.42	99.41	99.40	99.40	99.38	99.41	99.42	99.40	99.40	99.40	99.41	99.38	99.41
**B97**	95.10	95.09	95.15	99.52	99.52	99.52	99.51	99.46	99.51	99.50	99.51	99.53	99.52	99.51	99.49	99.51
**B104**	95.14	95.14	95.21	99.55	99.57	99.57	99.57	99.54	99.56	99.55	99.56	99.56	99.56	99.54	99.52	99.56
**B139**	95.12	95.13	95.18	99.52	99.56	99.54	99.54	99.48	99.54	99.50	99.55	99.55	99.55	99.52	99.49	99.54
**B144**	95.11	95.10	95.17	99.52	99.53	99.53	99.55	99.47	99.52	99.50	99.52	99.54	99.53	99.52	99.50	99.53
**B130**	95.12	95.12	95.18	99.54	99.57	99.56	99.55	99.51	99.53	99.52	99.56	99.53	99.55	99.52	99.51	99.52
**B137**	95.13	95.13	95.19	99.59	99.54	99.53	99.53	99.50	99.54	99.52	99.53	99.54	99.54	99.58	99.54	99.54
**B119**	95.10	95.10	95.15	99.52	99.52	99.54	99.53	99.46	99.52	99.49	99.51	99.52	99.54	99.51	99.50	99.52
**B107**	95.14	95.14	95.21	99.55	99.58	99.58	99.60	99.55	99.56	99.57	99.58	99.57	99.57	99.54	99.54	99.56
**B150**	95.20	95.19	95.27	99.42	99.41	99.42	99.40	99.38	99.41	99.42	99.41	99.41	99.40	99.42	99.40	99.40
**B94**	95.12	95.11	95.18	99.55	99.50	99.51	99.51	99.48	99.50	99.51	99.50	99.51	99.49	99.55	99.52	99.51
**B106**	95.11	95.12	95.18	99.53	99.52	99.51	99.51	99.45	99.51	99.47	99.51	99.52	99.51	99.53	99.50	99.52
**B115**	95.11	95.11	95.17	99.57	99.52	99.51	99.51	99.46	99.52	99.48	99.51	99.52	99.49	99.58	99.52	99.52
**B122**	95.12	95.11	95.17	99.55	99.55	99.54	99.52	99.48	99.51	99.50	99.54	99.55	99.55	99.54	99.50	99.55
**B132**	95.11	95.11	95.17	99.55	99.56	99.54	99.54	99.51	99.54	99.53	99.55	99.55	99.55	99.56	99.52	99.55
**B100**	95.12	95.11	95.17	99.55	99.58	99.56	99.56	99.50	99.52	99.52	99.57	99.55	99.57	99.53	99.50	99.55
**B138**	95.13	95.14	95.20	99.56	99.59	99.58	99.58	99.55	99.56	99.57	99.59	99.57	99.58	99.55	99.54	99.57
**B124**	95.12	95.12	95.19	99.55	99.56	99.56	99.55	99.53	99.57	99.56	99.55	99.57	99.55	99.54	99.52	99.55
**B109**	95.11	95.09	95.16	99.54	99.53	99.55	99.55	99.46	99.51	99.49	99.53	99.53	99.54	99.52	99.49	99.52
**B117**	95.12	95.13	95.19	99.55	99.57	99.56	99.55	99.54	99.55	99.55	99.57	99.57	99.55	99.54	99.52	99.56
**B116**	95.20	95.20	95.28	99.43	99.42	99.43	99.41	99.38	99.42	99.42	99.42	99.41	99.40	99.42	99.40	99.42
**B105**	95.12	95.13	95.19	99.55	99.57	99.56	99.55	99.54	99.55	99.55	99.57	99.57	99.55	99.54	99.52	99.56
**B133**	95.12	95.12	95.18	99.56	99.55	99.56	99.54	99.52	99.57	99.56	99.55	99.55	99.55	99.54	99.54	99.74
**B136**	95.11	95.10	95.17	99.55	99.56	99.55	99.56	99.49	99.52	99.51	99.55	99.55	99.56	99.52	99.50	99.54
**B118**	95.13	95.14	95.20	99.59	99.53	99.53	99.53	99.51	99.53	99.52	99.53	99.53	99.54	99.57	99.53	99.54
**B114**	95.12	95.10	95.17	99.51	99.52	99.53	99.53	99.46	99.52	99.46	99.51	99.52	99.51	99.52	99.49	99.51
**B125**	95.10	95.09	95.15	99.54	99.49	99.49	99.49	99.42	99.49	99.44	99.49	99.49	99.48	99.54	99.84	99.50
**B149**	95.26	95.26	95.34	99.36	99.34	99.34	99.34	99.31	99.34	99.34	99.34	99.34	99.33	99.35	99.33	99.34
**B151**	95.13	95.14	95.20	99.54	99.56	99.56	99.56	99.53	99.55	99.55	99.56	99.55	99.57	99.53	99.51	99.54

Table S2. Continued (page 3)

	B91	B93	B101	B108	B147	B97	B104	B139	B144	B130	B137	B119	B107	B150	B94	B106
**B10**	95.09	95.11	95.16	95.10	95.19	95.16	95.13	95.12	95.12	95.16	95.08	95.15	95.10	95.16	95.10	95.17
**B12**	95.18	95.21	95.20	95.19	95.30	95.19	95.22	95.19	95.20	95.19	95.18	95.19	95.21	95.27	95.20	95.21
**B19**	95.06	95.08	95.14	95.07	95.17	95.11	95.09	95.08	95.09	95.14	95.05	95.14	95.07	95.15	95.08	95.17
**B21**	95.17	95.19	95.19	95.18	95.29	95.19	95.21	95.19	95.19	95.19	95.16	95.18	95.19	95.25	95.20	95.21
**B27**	95.21	95.22	95.22	95.21	95.31	95.21	95.23	95.22	95.21	95.21	95.21	95.21	95.21	95.30	95.21	95.23
**B28**	94.86	94.86	94.87	94.86	94.95	94.87	94.89	94.88	94.87	94.86	94.85	94.87	94.87	94.91	94.88	94.89
**B33**	95.17	95.20	95.19	95.18	95.29	95.18	95.21	95.18	95.18	95.19	95.18	95.18	95.20	95.26	95.18	95.21
**B34**	95.21	95.22	95.22	95.22	95.31	95.22	95.23	95.22	95.22	95.22	95.21	95.21	95.21	95.30	95.22	95.23
**B37**	95.21	95.21	95.22	95.22	95.31	95.22	95.23	95.22	95.22	95.22	95.21	95.21	95.21	95.30	95.22	95.23
**B42**	95.21	95.22	95.22	95.21	95.32	95.21	95.23	95.21	95.21	95.21	95.20	95.21	95.22	95.29	95.22	95.22
**B44**	95.13	95.15	95.17	95.15	95.24	95.16	95.17	95.16	95.16	95.16	95.12	95.15	95.14	95.20	95.16	95.18
**B48**	95.18	95.20	95.20	95.20	95.30	95.19	95.22	95.20	95.19	95.20	95.18	95.19	95.21	95.27	95.20	95.21
**B49**	95.11	95.16	95.17	95.14	95.23	95.17	95.17	95.15	95.16	95.16	95.10	95.16	95.13	95.19	95.14	95.18
**B51**	95.14	95.15	95.17	95.14	95.25	95.16	95.16	95.16	95.16	95.16	95.13	95.15	95.15	95.22	95.17	95.18
**B52**	95.13	95.14	95.14	95.13	95.23	95.14	95.16	95.14	95.14	95.14	95.12	95.13	95.14	95.20	95.14	95.15
**B160**	95.10	95.13	95.12	95.10	95.19	95.11	95.13	95.12	95.11	95.11	95.10	95.11	95.13	95.16	95.10	95.13
**B163**	95.08	95.09	95.11	95.09	95.18	95.10	95.11	95.11	95.11	95.10	95.06	95.10	95.09	95.14	95.10	95.11
**B161**	95.12	95.14	95.14	95.12	95.21	95.13	95.16	95.13	95.13	95.14	95.12	95.13	95.14	95.18	95.13	95.15
**B165**	95.18	95.20	95.20	95.19	95.30	95.19	95.21	95.20	95.19	95.20	95.18	95.19	95.20	95.26	95.20	95.21
**B65**	99.47	99.48	99.51	99.47	99.34	99.48	99.49	99.48	99.46	99.50	99.48	99.50	99.48	99.34	99.48	99.51
**B66**	99.57	99.59	99.57	99.57	99.41	99.57	99.58	99.60	99.57	99.59	99.54	99.57	99.57	99.43	99.54	99.57
**B69**	99.53	99.59	99.56	99.56	99.37	99.53	99.55	99.53	99.55	99.57	99.49	99.55	99.53	99.40	99.50	99.53
**B77**	99.52	99.53	99.55	99.53	99.37	99.53	99.54	99.53	99.56	99.55	99.49	99.54	99.55	99.37	99.50	99.52
**B79**	99.55	99.56	99.56	99.56	99.38	99.54	99.56	99.55	99.55	99.57	99.52	99.54	99.55	99.40	99.51	99.53
**B81**	99.54	99.53	99.54	99.55	99.38	99.54	99.54	99.54	99.53	99.55	99.50	99.54	99.53	99.39	99.50	99.54
**B82**	99.57	99.59	99.57	99.56	99.42	99.57	99.56	99.57	99.57	99.58	99.53	99.56	99.56	99.43	99.54	99.55
**B83**	99.56	99.59	99.57	99.57	99.40	99.56	99.57	99.59	99.56	99.58	99.54	99.57	99.57	99.42	99.53	99.56
**B84**	99.53	99.56	99.56	99.55	99.37	99.54	99.54	99.56	99.55	99.56	99.51	99.55	99.53	99.39	99.52	99.54
**B86**	99.55	99.59	99.57	99.57	99.40	99.55	99.57	99.57	99.57	99.58	99.53	99.56	99.56	99.42	99.52	99.55
**B87**	99.46	99.48	99.49	99.48	99.32	99.48	99.47	99.46	99.45	99.48	99.47	99.49	99.46	99.35	99.49	99.51
**B88**	99.48	99.49	99.49	99.51	99.34	99.50	99.48	99.48	99.48	99.50	99.46	99.50	99.48	99.35	99.50	99.50
**B90**	99.56	99.57	99.56	99.57	99.40	99.57	99.56	99.57	99.55	99.57	99.54	99.58	99.55	99.41	99.53	99.56
**B91**	*	99.56	99.55	99.56	99.39	99.88	99.54	99.55	99.56	99.55	99.51	99.55	99.56	99.41	99.52	99.54
**B93**	99.56	*	99.58	99.57	99.41	99.57	99.58	99.57	99.57	99.58	99.53	99.58	99.57	99.43	99.52	99.56
**B101**	99.50	99.54	*	99.53	99.36	99.52	99.56	99.54	99.54	99.55	99.46	99.53	99.54	99.38	99.48	99.52
**B108**	99.55	99.56	99.55	*	99.38	99.56	99.55	99.55	99.54	99.56	99.52	99.55	99.57	99.42	99.51	99.54
**B147**	99.40	99.42	99.41	99.41	*	99.41	99.41	99.41	99.41	99.41	99.42	99.39	99.39	99.51	99.40	99.40
**B97**	99.83	99.53	99.52	99.53	99.34	*	99.51	99.52	99.53	99.52	99.46	99.53	99.54	99.38	99.50	99.52
**B104**	99.55	99.57	99.59	99.57	99.40	99.55	*	99.56	99.57	99.57	99.53	99.55	99.55	99.43	99.52	99.55
**B139**	99.53	99.56	99.55	99.53	99.38	99.53	99.55	*	99.54	99.55	99.48	99.55	99.53	99.38	99.51	99.52
**B144**	99.55	99.54	99.54	99.53	99.39	99.55	99.54	99.53	*	99.53	99.46	99.52	99.53	99.39	99.49	99.51
**B130**	99.52	99.55	99.57	99.54	99.36	99.53	99.54	99.55	99.53	*	99.50	99.55	99.54	99.39	99.49	99.52
**B137**	99.53	99.54	99.53	99.55	99.41	99.53	99.53	99.53	99.52	99.54	*	99.52	99.54	99.43	99.54	99.55
**B119**	99.51	99.55	99.53	99.52	99.34	99.53	99.53	99.53	99.51	99.54	99.46	*	99.52	99.35	99.48	99.52
**B107**	99.58	99.58	99.58	99.60	99.40	99.58	99.57	99.56	99.56	99.57	99.54	99.56	*	99.42	99.53	99.55
**B150**	99.40	99.42	99.43	99.42	99.49	99.41	99.42	99.41	99.42	99.41	99.42	99.40	99.40	*	99.41	99.41
**B94**	99.52	99.50	99.51	99.51	99.37	99.52	99.51	99.51	99.50	99.50	99.51	99.51	99.49	99.40	*	99.52
**B106**	99.51	99.53	99.51	99.53	99.35	99.52	99.51	99.50	99.49	99.51	99.49	99.51	99.49	99.36	99.50	*
**B115**	99.51	99.51	99.51	99.53	99.40	99.52	99.50	99.51	99.50	99.52	99.75	99.51	99.50	99.42	99.53	99.54
**B122**	99.52	99.55	99.54	99.54	99.37	99.53	99.54	99.53	99.52	99.54	99.48	99.53	99.54	99.38	99.51	99.53
**B132**	99.54	99.58	99.55	99.55	99.39	99.54	99.54	99.54	99.53	99.55	99.54	99.54	99.54	99.40	99.52	99.56
**B100**	99.55	99.56	99.54	99.56	99.38	99.55	99.54	99.53	99.53	99.56	99.48	99.54	99.55	99.37	99.50	99.52
**B138**	99.56	99.59	99.58	99.57	99.42	99.57	99.57	99.58	99.57	99.58	99.54	99.56	99.56	99.43	99.54	99.56
**B124**	99.56	99.56	99.55	99.56	99.39	99.56	99.55	99.56	99.54	99.57	99.52	99.57	99.54	99.41	99.53	99.56
**B109**	99.52	99.53	99.54	99.53	99.37	99.52	99.54	99.53	99.52	99.54	99.45	99.52	99.53	99.38	99.47	99.52
**B117**	99.57	99.57	99.56	99.57	99.40	99.57	99.56	99.57	99.55	99.56	99.53	99.56	99.56	99.41	99.52	99.54
**B116**	99.42	99.42	99.42	99.43	99.49	99.42	99.42	99.41	99.42	99.43	99.42	99.42	99.40	99.53	99.42	99.42
**B105**	99.58	99.57	99.56	99.57	99.40	99.58	99.55	99.57	99.55	99.56	99.53	99.56	99.57	99.42	99.52	99.54
**B133**	99.58	99.56	99.55	99.57	99.40	99.59	99.56	99.55	99.56	99.55	99.53	99.57	99.54	99.40	99.52	99.55
**B136**	99.53	99.55	99.55	99.55	99.38	99.53	99.54	99.53	99.51	99.56	99.47	99.54	99.54	99.38	99.49	99.52
**B118**	99.53	99.54	99.53	99.55	99.41	99.53	99.53	99.53	99.52	99.53	99.86	99.52	99.53	99.42	99.54	99.55
**B114**	99.51	99.52	99.53	99.51	99.35	99.53	99.52	99.52	99.53	99.54	99.44	99.52	99.50	99.36	99.49	99.50
**B125**	99.48	99.49	99.50	99.51	99.34	99.50	99.48	99.48	99.48	99.50	99.47	99.51	99.48	99.35	99.50	99.51
**B149**	99.33	99.35	99.35	99.35	99.46	99.34	99.35	99.34	99.35	99.34	99.35	99.34	99.33	99.50	99.34	99.33
**B151**	99.53	99.57	99.56	99.55	99.39	99.54	99.56	99.56	99.55	99.57	99.53	99.55	99.55	99.41	99.51	99.53

Table S2. Continued (page 4)

	B115	B122	B132	B100	B138	B124	B109	B117	B116	B105	B133	B136	B118	B114	B125	B149	B151
**B10**	95.11	95.11	95.07	95.12	95.06	95.09	95.16	95.09	95.19	95.10	95.08	95.15	95.08	95.17	95.12	95.23	95.08
**B12**	95.19	95.19	95.17	95.21	95.17	95.20	95.20	95.18	95.28	95.18	95.18	95.20	95.18	95.22	95.18	95.33	95.18
**B19**	95.08	95.08	95.04	95.09	95.04	95.07	95.14	95.07	95.16	95.07	95.05	95.14	95.06	95.15	95.10	95.21	95.06
**B21**	95.19	95.18	95.14	95.19	95.14	95.18	95.19	95.18	95.29	95.18	95.16	95.19	95.17	95.21	95.18	95.32	95.16
**B27**	95.21	95.21	95.20	95.22	95.20	95.22	95.22	95.21	95.30	95.21	95.20	95.22	95.22	95.23	95.20	95.36	95.22
**B28**	94.85	94.87	94.84	94.88	94.85	94.87	94.88	94.87	94.94	94.87	94.86	94.88	94.85	94.88	94.86	94.96	94.85
**B33**	95.18	95.18	95.16	95.19	95.16	95.18	95.20	95.18	95.28	95.18	95.16	95.19	95.18	95.21	95.17	95.33	95.17
**B34**	95.21	95.21	95.20	95.23	95.21	95.21	95.22	95.21	95.30	95.21	95.20	95.22	95.21	95.23	95.20	95.36	95.23
**B37**	95.21	95.21	95.20	95.22	95.20	95.21	95.22	95.21	95.30	95.21	95.20	95.22	95.21	95.23	95.20	95.36	95.22
**B42**	95.20	95.21	95.19	95.22	95.20	95.22	95.22	95.21	95.30	95.21	95.21	95.22	95.20	95.23	95.20	95.35	95.21
**B44**	95.15	95.15	95.11	95.16	95.09	95.15	95.18	95.13	95.23	95.13	95.13	95.17	95.12	95.19	95.15	95.26	95.11
**B48**	95.19	95.19	95.17	95.21	95.17	95.20	95.20	95.18	95.28	95.18	95.18	95.20	95.19	95.22	95.18	95.33	95.19
**B49**	95.15	95.14	95.09	95.15	95.08	95.12	95.17	95.13	95.21	95.13	95.11	95.16	95.11	95.19	95.15	95.25	95.09
**B51**	95.15	95.16	95.12	95.17	95.11	95.15	95.17	95.15	95.24	95.15	95.14	95.17	95.13	95.18	95.15	95.28	95.13
**B52**	95.13	95.13	95.12	95.14	95.13	95.14	95.14	95.13	95.22	95.13	95.13	95.14	95.13	95.15	95.12	95.27	95.14
**B160**	95.10	95.11	95.09	95.12	95.09	95.11	95.12	95.11	95.18	95.11	95.10	95.12	95.10	95.13	95.10	95.22	95.10
**B163**	95.08	95.09	95.05	95.11	95.04	95.09	95.11	95.08	95.17	95.08	95.08	95.10	95.06	95.12	95.09	95.21	95.06
**B161**	95.12	95.13	95.11	95.15	95.10	95.14	95.14	95.13	95.20	95.12	95.11	95.14	95.13	95.15	95.12	95.24	95.12
**B165**	95.19	95.19	95.16	95.20	95.17	95.20	95.20	95.18	95.28	95.19	95.18	95.20	95.18	95.22	95.18	95.33	95.18
**B65**	99.50	99.51	99.45	99.52	99.44	99.49	99.53	99.47	99.35	99.47	99.49	99.52	99.47	99.50	99.51	99.28	99.44
**B66**	99.53	99.58	99.57	99.61	99.58	99.58	99.59	99.58	99.44	99.58	99.56	99.59	99.54	99.58	99.54	99.36	99.58
**B69**	99.50	99.54	99.52	99.56	99.53	99.55	99.55	99.53	99.40	99.53	99.54	99.56	99.49	99.56	99.52	99.33	99.55
**B77**	99.48	99.53	99.51	99.57	99.51	99.53	99.57	99.52	99.38	99.52	99.51	99.57	99.49	99.55	99.50	99.31	99.52
**B79**	99.51	99.55	99.54	99.57	99.54	99.56	99.56	99.56	99.41	99.55	99.53	99.57	99.52	99.57	99.52	99.33	99.55
**B81**	99.51	99.53	99.51	99.55	99.51	99.57	99.55	99.52	99.40	99.52	99.55	99.55	99.50	99.55	99.52	99.32	99.53
**B82**	99.53	99.55	99.56	99.59	99.56	99.57	99.58	99.56	99.44	99.56	99.57	99.58	99.53	99.56	99.53	99.36	99.57
**B83**	99.53	99.57	99.57	99.60	99.57	99.58	99.60	99.57	99.44	99.58	99.56	99.59	99.53	99.57	99.54	99.35	99.58
**B84**	99.50	99.56	99.53	99.58	99.51	99.56	99.58	99.54	99.40	99.54	99.53	99.58	99.50	99.55	99.52	99.32	99.52
**B86**	99.53	99.57	99.56	99.58	99.56	99.57	99.57	99.56	99.42	99.56	99.56	99.58	99.53	99.57	99.53	99.35	99.59
**B87**	99.51	99.48	99.46	99.48	99.42	99.47	99.50	99.47	99.35	99.47	99.46	99.50	99.46	99.48	99.52	99.28	99.42
**B88**	99.48	99.48	99.46	99.49	99.45	99.49	99.50	99.50	99.37	99.50	99.50	99.50	99.45	99.50	99.84	99.28	99.44
**B90**	99.53	99.58	99.56	99.57	99.55	99.56	99.57	99.57	99.43	99.57	99.74	99.58	99.53	99.56	99.54	99.35	99.55
**B91**	99.52	99.54	99.54	99.57	99.53	99.57	99.56	99.55	99.42	99.56	99.57	99.56	99.51	99.55	99.52	99.33	99.54
**B93**	99.53	99.57	99.59	99.59	99.57	99.57	99.58	99.57	99.44	99.57	99.55	99.59	99.53	99.57	99.54	99.36	99.58
**B101**	99.49	99.52	99.50	99.54	99.50	99.52	99.55	99.52	99.39	99.52	99.51	99.55	99.46	99.55	99.50	99.30	99.50
**B108**	99.52	99.56	99.54	99.58	99.53	99.55	99.57	99.56	99.41	99.56	99.55	99.58	99.52	99.55	99.53	99.34	99.53
**B147**	99.42	99.40	99.40	99.41	99.40	99.41	99.42	99.41	99.50	99.41	99.41	99.42	99.41	99.40	99.39	99.47	99.40
**B97**	99.48	99.51	99.48	99.53	99.48	99.51	99.53	99.53	99.38	99.53	99.53	99.53	99.46	99.53	99.49	99.30	99.47
**B104**	99.52	99.55	99.56	99.57	99.55	99.56	99.58	99.55	99.43	99.55	99.56	99.57	99.52	99.58	99.53	99.35	99.57
**B139**	99.50	99.53	99.50	99.55	99.50	99.55	99.55	99.55	99.40	99.55	99.53	99.55	99.47	99.55	99.49	99.32	99.51
**B144**	99.47	99.53	99.49	99.54	99.50	99.53	99.55	99.53	99.39	99.53	99.54	99.54	99.46	99.55	99.50	99.32	99.50
**B130**	99.49	99.54	99.52	99.56	99.52	99.54	99.56	99.53	99.39	99.53	99.51	99.57	99.49	99.56	99.51	99.32	99.54
**B137**	99.78	99.53	99.55	99.54	99.52	99.54	99.54	99.54	99.43	99.54	99.53	99.55	99.85	99.53	99.54	99.36	99.53
**B119**	99.49	99.51	99.48	99.54	99.48	99.53	99.53	99.51	99.37	99.51	99.52	99.54	99.46	99.53	99.51	99.29	99.49
**B107**	99.53	99.56	99.56	99.57	99.55	99.57	99.57	99.57	99.43	99.58	99.55	99.58	99.54	99.57	99.54	99.35	99.57
**B150**	99.43	99.40	99.40	99.41	99.41	99.40	99.43	99.40	99.52	99.41	99.39	99.42	99.41	99.41	99.40	99.50	99.41
**B94**	99.51	99.52	99.51	99.51	99.49	99.53	99.51	99.48	99.40	99.48	99.50	99.51	99.50	99.53	99.53	99.34	99.50
**B106**	99.51	99.52	99.51	99.52	99.48	99.54	99.53	99.49	99.37	99.49	99.51	99.52	99.49	99.51	99.51	99.29	99.47
**B115**	*	99.51	99.52	99.52	99.49	99.52	99.52	99.51	99.43	99.51	99.52	99.52	99.74	99.51	99.53	99.35	99.48
**B122**	99.48	*	99.50	99.57	99.51	99.54	99.56	99.54	99.39	99.54	99.54	99.57	99.47	99.54	99.50	99.32	99.50
**B132**	99.54	99.54	*	99.56	99.53	99.55	99.55	99.55	99.42	99.55	99.55	99.56	99.54	99.54	99.53	99.34	99.55
**B100**	99.50	99.56	99.51	*	99.52	99.56	99.63	99.56	99.40	99.55	99.54	99.64	99.47	99.56	99.50	99.31	99.54
**B138**	99.54	99.57	99.56	99.58	*	99.57	99.58	99.57	99.44	99.56	99.56	99.59	99.54	99.58	99.54	99.36	99.59
**B124**	99.51	99.54	99.54	99.56	99.53	*	99.56	99.55	99.41	99.55	99.54	99.56	99.51	99.56	99.52	99.33	99.55
**B109**	99.47	99.56	99.48	99.63	99.49	99.54	*	99.52	99.39	99.52	99.52	99.65	99.45	99.55	99.49	99.31	99.48
**B117**	99.52	99.57	99.54	99.58	99.55	99.57	99.57	*	99.42	99.85	99.56	99.58	99.52	99.57	99.53	99.34	99.55
**B116**	99.43	99.41	99.42	99.43	99.41	99.41	99.44	99.41	*	99.41	99.42	99.43	99.41	99.42	99.41	99.50	99.41
**B105**	99.52	99.57	99.54	99.58	99.54	99.57	99.57	99.85	99.42	*	99.55	99.58	99.52	99.56	99.53	99.34	99.55
**B133**	99.52	99.56	99.55	99.56	99.54	99.56	99.56	99.56	99.43	99.55	*	99.57	99.53	99.55	99.54	99.34	99.54
**B136**	99.49	99.56	99.50	99.64	99.51	99.55	99.66	99.54	99.39	99.54	99.54	*	99.47	99.56	99.50	99.31	99.50
**B118**	99.77	99.53	99.56	99.54	99.52	99.54	99.54	99.54	99.43	99.54	99.53	99.54	*	99.52	99.54	99.35	99.52
**B114**	99.47	99.50	99.46	99.54	99.48	99.53	99.55	99.52	99.36	99.51	99.51	99.55	99.43	*	99.50	99.28	99.50
**B125**	99.48	99.48	99.47	99.49	99.46	99.50	99.50	99.51	99.38	99.51	99.51	99.51	99.46	99.50	*	99.28	99.44
**B149**	99.36	99.34	99.34	99.35	99.33	99.33	99.36	99.34	99.50	99.34	99.34	99.35	99.34	99.34	99.33	*	99.34
**B151**	99.51	99.55	99.55	99.59	99.56	99.56	99.56	99.54	99.41	99.54	99.54	99.56	99.52	99.59	99.51	99.33	*

Table S3. DBCAN annotations of genes unique to *Lonsdalea quercina* Colorado and California populations

Population	Query	Enzyme*	Substrate
Colorado	All2_02409	GT51	peptidoglycan
Colorado	All2_02530	GH37	trehalose
Colorado	All2_03014	GH23	peptidoglycan
California	All2_00837	GH37	trehalose
California	All2_01181	GH12	xyloglucan
California	All2_02137	GT51	peptidoglycan
California	All2_02501	GH23	peptidoglycan

GT - glycosyl transferase family

GH - glycosyl hydrolase family

Table S4. PHI-base annotations of genes unique to *Lonsdalea quercina* Colorado and California populations

Population	Query	Protein accession	PHI accession	Gene name	Tax ID
Colorado	All2_00061	Q8Z4N6	609__616	SseA	28901
Colorado	All2_00184	C9Z1K8	11107	SCAB_85461	1930
Colorado	All2_00292	Q4ZTI4	7699	Smp	317
Colorado	All2_00515	P33221	8653	PurT	562
Colorado	All2_00572	A0A0F6B5T1	10085	STM14_RS15360	28901
Colorado	All2_00573	A0A0F6B5T0	10084	STM14_RS15355	28901
Colorado	All2_00574	A0A0F6B5S9	10083	STM14_RS15350	28901
Colorado	All2_00575	A0A0F6B5S8	10082	STM14_RS15345	28901
Colorado	All2_00585	A0A0H3NC59	9496	YeaB	28901
Colorado	All2_00590	A0A165G9C3	6870	YeaZ	669
Colorado	All2_00907	Q8XW15	8884	AroG1	305
Colorado	All2_00931	Q8ZFR1	8898	CobB_(Yp_1760)	632
Colorado	All2_01589	P0ADC1	10313	LptE	562
Colorado	All2_01598	A1JQ74	7671	YbeY	630
Colorado	All2_01708	Q66FK0	7909	HmuV	633
Colorado	All2_02005	P13738	3266	NhaA	562
Colorado	All2_02442	Q8ZQ79	8363__9965	SseI__SrfH	28901
Colorado	All2_02530	P13482	10199	TreA	562
Colorado	All2_02588	P0AFV4	11093	MepS	562
Colorado	All2_02901	Q8CWG4	3272	GlpD	632
Colorado	All2_02991	P07822	10989	FhuD	562
Colorado	All2_03062	A0A0F6BB74	6981	Tsr	28901
Colorado	All2_03071	Q9K033	8623	Cab_(NMB0789)	487
Colorado	All2_03114	E0SLG2	8567	HdfR_(Dda3937_04419)	204038
California	All2_00172	P0ADC1	10313	LptE	562
California	All2_00488	Q8XW15	8884	AroG1	305
California	All2_00163	A1JQ74	7671	YbeY	630
California	All2_03080	E0SLG2	8567	HdfR_(Dda3937_04419)	204038
California	All2_00463	Q8ZFR1	8898	CobB_(Yp_1760)	632

Table S4. Continued (page 2)

California	All2_00896	P0AFV4	11093	MepS	562
California	All2_03014	B4E5A2	3524__9576	PaaA	95486
California	All2_03098	Q8Z4N6	609__616	SseA	28901
California	All2_02954	A0A0F6BB74	6981	Tsr	28901
California	All2_02258	A0A0H3NC59	9496	YeaB	28901
California	All2_02333	P39172	11005	ZnuA	562
California	All2_02253	A0A165G9C3	6870	YeaZ	669
California	All2_01274	P13738	3266	NhaA	562
California	All2_02822	H7C7L2	7821	OppF_(bb0335)	139
California	All2_03023	Q4UZG1	3941	XC_0481	339
California	All2_01707	C9Z1K8	11107	SCAB_85461	1930
California	All2_01200	A0A0U1YU94	6362	PilA	553
California	All2_02324	P33221	8653	PurT	562
California	All2_00837	P13482	10199	TreA	562
California	All2_02810	Q8CWG4	3272	GlpD	632
California	All2_02268	A0A0F6B5S8	10082	STM14_RS15345	28901
California	All2_02270	A0A0F6B5T0	10084	STM14_RS15355	28901
California	All2_02269	A0A0F6B5S9	10083	STM14_RS15350	28901
California	All2_00055	Q66FK0	7909	HmuV	633
California	All2_02522	P07822	10989	FhuD	562
California	All2_02271	A0A0F6B5T1	10085	STM14_RS15360	28901

Table S4. Continued (page 3)

Population	Phenotype	evalue	% identity
Colorado	reduced_virulence	3.00E-116	60.58
Colorado	reduced_virulence	3.00E-22	53.12
Colorado	unaffected_pathogenicity	8.00E-80	52.17
Colorado	reduced_virulence/unaffected_pathogenicity	0	76.79
Colorado	unaffected_pathogenicity	1.00E-94	51.67
Colorado	unaffected_pathogenicity	4.00E-134	65.25
Colorado	unaffected_pathogenicity	1.00E-113	59.5
Colorado	unaffected_pathogenicity	3.00E-139	65.02
Colorado	reduced_virulence	3.00E-71	59.24
Colorado	reduced_virulence	1.00E-89	57.51
Colorado	loss_of_pathogenicity	4.00E-132	53.56
Colorado	reduced_virulence	9.00E-143	73
Colorado	increased_virulence/unaffected_pathogenicity	2.00E-66	58.43
Colorado	reduced_virulence	8.00E-92	83.12
Colorado	unaffected_pathogenicity	2.00E-98	56.65
Colorado	reduced_virulence	1.00E-125	57.18
Colorado	effector	2.00E-36	50.79
Colorado	reduced_virulence/unaffected_pathogenicity	0	56.96
Colorado	reduced_virulence	2.00E-95	68.78
Colorado	unaffected_pathogenicity	0	77.15
Colorado	unaffected_pathogenicity	2.00E-106	54.7
Colorado	reduced_virulence	0	62.48
Colorado	unaffected_pathogenicity	1.00E-83	52.42
Colorado	reduced_virulence	7.00E-150	76.92
California	increased_virulence/unaffected_pathogenicity	7.00E-67	56.9
California	loss_of_pathogenicity	9.00E-134	54.13
California	reduced_virulence	8.00E-89	81.17
California	reduced_virulence	3.00E-146	76.47
California	reduced_virulence	4.00E-139	71.86

Table S4. Continued (page 4)

California	reduced_virulence	1.00E-96	68.78
California	reduced_virulence	2.00E-147	63.49
California	reduced_virulence	1.00E-118	60.65
California	reduced_virulence	0	60.39
California	reduced_virulence	3.00E-72	59.34
California	reduced_virulence	3.00E-137	58.2
California	reduced_virulence	1.00E-88	56.22
California	reduced_virulence	5.00E-119	55.7
California	reduced_virulence	3.00E-77	53.33
California	reduced_virulence	2.00E-71	53.16
California	reduced_virulence	2.00E-22	53.12
California	reduced_virulence	6.00E-49	52.41
California	reduced_virulence/unaffected_pathogenicity	0	77.55
California	reduced_virulence/unaffected_pathogenicity	0	56.9
California	unaffected_pathogenicity	0	76.51
California	unaffected_pathogenicity	3.00E-142	66.08
California	unaffected_pathogenicity	2.00E-131	65.11
California	unaffected_pathogenicity	4.00E-117	61.71
California	unaffected_pathogenicity	1.00E-97	56.86
California	unaffected_pathogenicity	8.00E-105	54.01
California	unaffected_pathogenicity	1.00E-95	51.12
